# Success and Self-Doubt: Prevalence and Predictors of Imposter Phenomenon in a Cohort of Medical Students from Saudi Arabia

**DOI:** 10.3390/healthcare13233172

**Published:** 2025-12-04

**Authors:** Sarah Alammar, Hala A. Mahmoud, Salma A. Metwally, Maryam Abdulla, Tanya Almutairi, Hala Tamim, Homoud Batel Alabri, Noara Alhusseini, Muhammad Raihan Sajid

**Affiliations:** 1 College of Medicine, Alfaisal University, Riyadh 11533, Saudi Arabia; salammar@alfaisal.edu (S.A.); hamahmoud@alfaisal.edu (H.A.M.); smetwally@alfaisal.edu (S.A.M.); mabdulla@alfaisal.edu (M.A.); talmuairi@alfaisal.edu (T.A.); hatamim@alfaisal.edu (H.T.); nalhusseini@alfaisal.edu (N.A.); 2 Department of Student Affairs, Alfaisal University, Riyadh 11533, Saudi Arabia; halabri@alfaisal.edu

**Keywords:** imposter phenomenon, imposter syndrome, medical students, perfectionism, Saudi Arabia

## Abstract

**Highlights:**

**What are the main findings**

- A high prevalence of Imposter Phenomenon (IP) was found among medical students, with nearly half (49.2%) reporting frequent IP characteristics.
- Perfectionism was identified as a strong, statistically significant positive predictor of IP severity in the student cohort.

**What are the implications of the main findings?**

- Interventions targeting maladaptive perfectionism and fostering self-compassion are needed to improve the mental well-being of medical students in this region.
- Unlike some global studies, academic performance (GPA) and year of study were not significant predictors of IP, suggesting institutional or cultural factors may be dominant.

**Abstract:**

**Background/Objectives**: Imposter Phenomenon (IP) describes internal doubt and fear of being exposed as fraud despite achievements. Coined by Clance and Imes, it is linked to societal pressures and attributing success to luck rather than ability. The main aim of this study was to investigate the prevalence and factors associated with IP among Alfaisal University medical students. **Methods**: A cross-sectional study was conducted using a self-reported questionnaire to assess IP and perfectionism in 295 medical students (74.9% female). Descriptive statistics and regression analyses were used to analyze the data. **Results**: Nearly half (49.2%) of the participants reported frequent imposter characteristics, with a substantial portion (27.1%) experiencing intense characteristics. Females were more likely to experience IP compared to males. Neither GPA nor year of study were significant predictors of IP. However, perfectionism scores showed a strong positive association with IP. **Conclusions**: This study identified a high prevalence of IP among Alfaisal medical students. While gender differences were observed, academic performance and year of study were not significant predictors. Perfectionism emerged as an associated factor of IP. These findings suggest that interventions targeting perfectionism and promoting self-compassion may benefit medical students experiencing IP. Further research is needed to explore the underlying causes of IP and its impact on student well-being.

## 1. Introduction

The Imposter Phenomenon (IP) is a psychological term describing an internal feeling of self-doubt and a fear of being exposed as a fraud despite various successes [1]. The concept of imposterism was initially established by Clance and Imes to describe a feeling of “intellectual phoniness” and generalized incompetence in the context of societal standards that exert pressure and family dynamics [2]. As a result of this phenomenon, people tend to attribute their successes to factors other than their intellect and hard work, such as mere luck or convenient relations and opportunities [3,4,5]. The inability to internalize success has been correlated with various psychological factors, including maladaptive perfectionism, anxiety, and depression, with reports of burnout and low self-esteem despite evidence of competence [6,7,8,9,10]. IP is prevalent in fields with high levels of competition that require profound intellectual ability [11]. This naturally manifests in university students due to the competitive nature of higher education [12], leading to the persistence of stress, self-doubt, and low self-confidence [12,13].

Medicine is a profession that attracts high-achieving and ambitious students who tend to be highly perfectionistic [14]. This poses a considerable risk of this demographic experiencing IP due to the high expectations in the field [15]. In the context of healthcare, global literature suggests that the prevalence of the Imposter Phenomenon among medical students and physicians ranges from 22% to 70.3% [4,13,16,17,18]. Additionally, the local prevalence of Imposter Phenomenon ranges from 7% to 60% [6,19,20]. Due to the high-achieving nature of the healthcare field, IP is associated with burnout and negative personality characteristics [2,21]. Furthermore, IP is linked with poor mental and physical health, hindered academic performance, and, in extreme cases, suicide [15,18,21].

With the current understanding that IP is a risk factor for declining psychological health, it is crucial to investigate potential predisposing factors contributing to these feelings of imposterism. One significant factor worth assessing is whether gender disparities contribute to an individual’s susceptibility to imposterism. A larger proportion of females experience clinical IP compared to males [22,23,24,25,26,27,28].

Another key factor is the stage of academic training. In Saudi Arabia, medical schools follow a six-year undergraduate program. The first three years make up the preclinical phase, which focuses on foundational sciences, while the last three years are the clinical phase, involving hospital rotations and direct patient care. As medical students advance in their training, greater accountability and pressures are placed on them. An American study examining medical students found that IP levels increase significantly toward the last years of study. The reason could be that they are approaching their first day as doctors and are under more pressure to prepare for the competitive matching process [16]. Another study suggests that clinicians at all levels experience IP feelings, which are exacerbated during periods of transition [29]. This relates to Clance’s theory that encountering novel challenges exacerbates imposter feelings [2]. However, other studies show no association between the progression of studies and IP, implying a static state of imposterism [30].

The high-achieving nature of medical school also serves as an interesting point of investigation regarding the potential association between grade point average (GPA) and imposterism. The competition and hierarchy presented in medical professions heighten a medical student’s fear of failure and their need to prove themselves worthy, which seems only to increase once they become physicians [31]. Current literature suggests that individuals who score high on the IP scale do not significantly differ in academic performance from those who score lower, suggesting no association between imposterism and GPA [12,20]. However, high IP scorers are more likely to face test performance anxiety and have decreased expectations regarding future achievements [12,32,33]. This serves as an important point of investigation due to the risk of future medical professionals being exposed to burnout and psychological distress that can negatively impact patient care [18,34,35].

Perfectionism, as described by Hollender [36], is the imposition of exceedingly high-performance standards beyond what is typically required in any given situation. Individuals exhibiting perfectionist tendencies may set unattainably high standards, harbor fears of failure, experience feelings of inadequacy, and engage in self-critical behavior [37]. Notably, studies have consistently demonstrated a significant link between perfectionism and the Imposter Phenomenon, with perfectionists more likely to experience imposter fears [38], as those experiencing the Imposter Phenomenon tend to disregard positive feedback and maintain strict standards for self-assessment [39]. It has been suggested that the Imposter Phenomenon may not be a product of high standards but instead is caused by what researchers refer to as the “perfectionist discrepancy,” which is the constant focus on one’s flaws [40]. Moreover, those who experience the Imposter Phenomenon are more likely to engage in maladaptive perfectionist behaviors, such as ruminating over mistakes and excessive planning [39]. This illustrates the hypothesized relationship between perfectionism and the imposter phenomenon.

While IP is recognized globally, there is limited data from Saudi Arabia examining its association with key predictors like perfectionism, gender, and academic progression within a single cohort using validated scales. Therefore, our study aims to address this gap by analyzing IP prevalence in medical students at Alfaisal University and its associations with gender, academic year, GPA, and perfectionism. We hypothesized that female gender, higher academic year, and greater perfectionism would be significant positive predictors of IP severity.

## 2. Methods

This study was designed as a cross-sectional study and conducted at the College of Medicine, Alfaisal University in Riyadh, Saudi Arabia, from November 2023 until May 2024. The study was advertised to all approximately 1200 medical students across all six years through official class representatives, WhatsApp groups and emails managed by Student Affairs. No direct access to student contact lists was granted; gatekeepers (class representatives and administrative staff) distributed the study advertisement. A dedicated email was provided for questions. The questionnaire was administered digitally via Google Forms. The first page contained the participant information sheet and digital consent form; participants had to select “I consent” to proceed. The questionnaire took approximately 7–10 min to complete. Bi-weekly reminders were sent to encourage participation. Convenience sampling was employed to recruit students from 1st to 6th-year medical school during the academic year 2023–2024. Inclusion criteria included students from all six years of medical school to identify our study population. Students from different colleges in Alfaisal University, besides the College of Medicine, were excluded.

### 2.1. Measures

A self-administered questionnaire comprising two main sections was distributed among Alfaisal medical students.

Demographics included gender, academic level, and cumulative GPA.

Clance Imposter Phenomenon Scale (CIPS): 20 items rated 1–5 (Not at all true to Very true). Total scores (20–100) categorized as: ≤40 (few characteristics), 41–60 (moderate), 61–80 (frequent), >80 (intense) [41]. The scale showed excellent internal consistency in our sample (Cronbach’s α = 0.92).

Perfectionism: Measured with a single item: “Do you consider yourself a perfectionist?” rated 1–5 (Strongly Disagree to Strongly Agree). While a multi-item scale would be ideal, single-item measures provide a practical alternative in brief surveys. It is important to note that this single item serves as a brief indicator rather than a comprehensive assessment using a validated multi-item scale.

GPA was categorized as “<3” and “≥3”. Academic year was grouped as “preclinical (Years 1–3)” and “clinical (Years 4–6)”. →This grouping reflects the college’s structure, where the preclinical cohort is typically larger. The gender distribution in our sample (approx. 75% female) reflects the overall composition of the College of Medicine.

CIPS was chosen due to its internal consistency reliability and item discrimination which was found to be satisfactory [42]. This structured psychometric instrument was used to assess the severity of imposter feelings among medical students by identifying feelings of self-doubt, failure to repeat success, and fear of being less capable than others. The CIPS comprises 20 items, each with a five-point scale, used to measure the severity of the imposter phenomenon, ranging from “none” to “very severe” (1–5). The total score could range from 20 to 100. A score of 40 or below indicated that the respondent exhibited few characteristics of the imposter phenomenon. A score between 41 and 60 suggested moderate experiences with the imposter phenomenon. Scores between 61 and 80 signaled frequent imposter feelings, and scores above 80 indicated that the respondent often experienced intense imposter phenomenon [41]. Participation was voluntary, confidential, and anonymous. No personally identifiable information, such as names or college IDs, was collected to ensure confidentiality. The research proposal was approved by the Institutional Review Board (IRB) at Alfaisal University (IRB-20246). The participants’ written consent was taken on the online questionnaire as per the IRB approval. A sample size calculator was used, and the sample size calculated was 306 participants to achieve a 95% confidence interval with a 5% margin of error. However, a convenient sample of 299 was achieved. This sample was able to detect a margin of error of 5.03%.

### 2.2. Statistical Analysis

Descriptive statistics were performed to describe the association of IP characteristics with gender, GPA, academic year, and perfectionism, and they were documented as percentages and frequencies. Missing data for CIPS was minimal; listwise deletion was applied in regression. Missing GPA data (*n* = 8) was excluded from GPA analyses. Analyses used SPSS v29 with significance at *p* < 0.05. A chi-square test was used to compare categorical variables. For unadjusted and adjusted analysis of the predictors of the development of imposter characteristics, linear regression, both single and multiple, was used. 

## 3. Results

**Figure 1:**
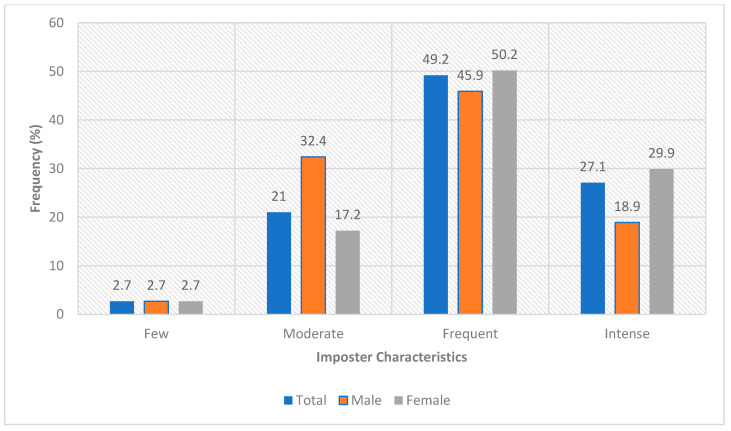
Frequency distribution of imposter characteristics among participants.

In total, 299 responses were collected, and after manually filtering the results that did not meet the inclusion criteria, 295 responses were included in the final analysis. Four were excluded (not medical students). The data in Figure 1 reveals that nearly half of the participants (49.2%) frequently displayed imposter characteristics, while a very small proportion (2.7%) reported few characteristics. Moderate and intense imposter characteristics are observed in a substantial portion of the sample.

Figure 1 depicts the percentage of participants categorized into four levels of imposterism: Few (2.7%), Moderate (21%), Frequent (49.2%), and Intense (27.1%).

A bivariate association of the participants’ characteristics with CIPS categories was conducted. As seen in Table 1, the respondents consisted of 221 (74.9%) females and 74 (25.1%) males. For GPA, eight respondents preferred not to state their GPA, leaving 287 responses, 43 (14.6%) of which had a GPA less than 3, and 244 (82.7%) with a GPA greater than or equal to 3. In terms of the clinical and preclinical phases of study, there were a total of 239 (81%) preclinical and 56 (18.9%) clinical students. The findings indicate that 8 (2.7%), 62 (21%), 145 (49.2%), and 80 (27.1%) medical students, respectively, exhibited few, moderate, frequent, and intense IP characteristics. Lastly, the mean and standard deviation of perfectionism were identified to compare significant mean differences among the varying levels of imposterism.

Table 1 describes the frequency of gender, GPA, academic year of study, and perfectionism with imposter characteristics. Results reveal that gender was significantly associated with imposter characteristics (*p* < 0.05). Additionally, the association between perfectionism and imposter characteristics was statistically significant (*p* < 0.05), with the difference being between moderate and frequent imposter characteristics, moderate and intense, and frequent and intense.

To establish the predictors of the development of imposter characteristics, a simple and multiple linear regression analysis was employed. As evidenced by the adjusted analysis, an increase in perfectionism by one unit was associated with an increase in IP by 2.5 (95% CI: 1.3 to 3.7, *p* < 0.001).

Table 2 linear regression was used to assess various predictors of the development of imposter characteristics.

## 4. Discussion

This study investigated the prevalence and factors associated with Imposter Phenomenon (IP) among Alfaisal medical students. We found a high prevalence of IP, with nearly half (49.2%) of the participants reporting frequent imposter characteristics and a substantial portion (27.1%) experiencing intense characteristics. This suggests that imposter phenomenon is a significant concern for Alfaisal medical students. Likewise, a study from Malaysia has reported a similar frequency of IP among medical students as at Alfaisal University [43]. These findings indicated that almost half of medical students have persistent self-doubt and worry about being exposed as academic fraudsters. However, several reported higher percentages of students experiencing imposter phenomenon. In a local Saudi study, almost 84% of dental students reported having IP [6]. Another study from Pakistan showed the frequency of 70% IP among dental students [44]. This aligns with some studies [43] but contrasts with others reporting higher prevalence [6,44]. These differences may be due to variations in sample characteristics, cultural factors, institutional environment, or measurement tools. This high prevalence is concerning, given the ample evidence linking IP to anxiety, depression, and low self-esteem. This could be explained, in part, by the fact that medical students are constantly under pressure to perform well since they are seen as high performers. Additionally, during internship, those with imposter characteristics are less confident in their ability to handle obstacles and are more likely to believe they should drop out of medical school.

Another significant finding was that females were more likely to experience imposter feelings compared to males. This aligns with previous research on IP prevalence in high-achieving populations [2,19,40,41,44]. The emergence of gender differences in our study may reflect specific cultural and institutional contexts that potentially exacerbate imposter feelings in female students. This indirect connection between IP and gender is hypothesized to be connected to neuroticism and self-esteem [10,37,45], where it was found that there is higher neuroticism and lower self-esteem in women compared to men [46]. In Saudi Arabia specifically, this lower self-confidence, resulting in more intense IP, could be attributed to the lack of exposure of females to the public, as seen in previously published studies [6]. Another possible reason for the increased IP in females is the cultural views on gender roles and female education, as well as environments with fewer female role models. Additionally, it was implied that straying from traditional gender roles may result in an internal conflict in high-achieving women [4,47]. However, several previously published articles have demonstrated no gender difference in relation to IP [43].

Regarding GPA and academic year, previously published studies showed that students in higher academic years were found to have a higher frequency of IP [16,43]. One study suggests that clinicians at all levels experience IP feelings, which would be exacerbated at periods of transitions [29]. This relates to Clance’s theory that encountering novel challenges exacerbates imposter feelings [2]. In contrast to some previous literature [16,29], our study found that neither academic year nor GPA were significant predictors of IP. This suggests that at our institution, feelings of imposterism may be a pervasive experience throughout the medical program, rather than being concentrated in specific transitional phases like the clinical years. The non-significant association with GPA aligns with other studies [12,20] and reinforces the core nature of IP as an internal experience of intellectual phoniness that is disconnected from objective academic achievement. This dissociation underscores that high performers are not immune to self-doubt. This suggests factors beyond academic progression dominate IP in this population. The competitive medical school environment may instill pervasive imposterism from the outset that remains relatively stable. This can be attributed to the fact that when students advance through several academic years, they not only encounter fresh difficulties, as indicated by Clance, but they also regard themselves as more capable, autonomous, and powerful. This suggests that factors beyond academic performance may contribute to IP in medical school. Several studies have shown similar findings as well [28,30,48].

Our analysis indicated a strong, statistically significant association between self-reported perfectionist tendencies and IP severity. This finding is consistent with a robust body of literature linking maladaptive perfectionism to IP [4,25,39]. Students who rated themselves higher on a perfectionism screening item were more likely to experience frequent or intense imposter characteristics. However, as perfectionism was assessed with a single item, this finding should be interpreted as preliminary. Future research using validated, multi-dimensional scales is necessary to delineate which specific aspects of perfectionism (e.g., self-critical evaluation, high personal standards) are most strongly driving this relationship in our population. Other studies have also highlighted that severe perfectionism raises the risk for anxiety and depressive disorders and can contribute to IP [4,25,39,40]. In one study, perfectionism, imposter phenomenon, and current psychological distress were found to be strongly associated with each program, and these personality traits were found to be more predictive of psychological adjustment than most of the demographic variables previously linked to psychological distress in students pursuing health professions [25].

### 4.1. Strengths of the Study

This is one of the first studies to explore IP in Saudi Arabia, specifically in Alfaisal medical students, providing valuable data for the institution. Furthermore, the use of validated measures for IP strengthens the research methodology.

### 4.2. Limitations and Future Directions

This study has several limitations. It relied on self-reported data, which is susceptible to information bias. Additionally, potential confounding factors such as ethnicity, socioeconomic status, and culture were not accounted for. The cross-sectional design further limits the ability to infer causality. A key limitation of this study is the measurement of perfectionism using a single-item indicator rather than a validated, multi-dimensional scale. While this provided an efficient screening measure, it prevents a nuanced understanding of the construct and likely oversimplifies its relationship with IP. The strong association observed should therefore be considered hypothesis-generating, warranting confirmation with more robust instruments, such as the Frost Multidimensional Perfectionism Scale. Gender imbalance reflecting institutional demographics but potentially limiting generalizability

Grouping of academic years potentially obscuring nuanced variations between levels.

Despite these limitations, the study provides valuable insights into the prevalence and factors associated with IP in medical students. However, further research is needed to explore its impact on academic performance and mental health among Alfaisal medical students.

## 5. Conclusions

This study identified high IP prevalence among Alfaisal medical students. While gender differences existed, academic performance and year were not significant predictors. Perfectionism was strongly associated with IP. These findings have important practical implications. Medical schools should develop targeted support programs including workshops and counseling services focused on combating maladaptive perfectionism, fostering self-compassion, and normalizing experiences of self-doubt. Further research should explore IP’s underlying causes and impact on mental health and academic performance. Addressing IP can significantly contribute to student well-being and success.

## Figures and Tables

**Table 1:** Bivariate Association of Study Participants’ Characteristics with CIPS Categories.

				CIPS		
	Total N(%)	Few N(%)	Moderate N(%)	Frequent N(%)	Intense N(%)	*p* Value
Total	295 (100.0)	8 (2.7)	62 (21.0)	145 (49.2)	80 (27.1)	
Gender						
Female	221 (74.9)	6 (2.7)	38 (17.2)	111 (50.2)	66 (29.9)	0.032
Male	74 (25.1)	2 (2.7)	24 (32.4)	34 (45.9)	14 (18.9)	
GPA						
<3	43 (14.6)	3 (7.0)	7 (16.3)	23 (53.5)	10 (23.3)	0.229
≥3	244 (82.7)	5 (2.0)	55 (22.5)	117 (48.0)	67 (27.5)	
Missing	8 (2.7)					
Year						
Preclinical (Years 1–3)	239 (81.0)	5 (2.1)	50 (20.9)	120 (50.2)	64 (26.8)	0.546
Clinical (Years 4–6)	56 (18.9)	3 (5.4)	12 (21.4)	25 (44.6)	16 (28.6)	
Perfectionist						
Mean (SD)	3.6 (1.4)	3.1 (1.6)	3.0 (1.4)	3.6 (1.3)	4.0 (1.3)	<0.001 ^a^

^a^ Difference is between moderate and frequent, moderate and intense, and frequent and intense.

**Table 2:** Shows results of regression analyses. In adjusted analysis, each one-unit increase in perfectionism was associated with a 2.5-point increase in IP score (95% CI: 1.3 to 3.7, *p* < 0.001).

	Simple Linear Regression (SLR)			Multiple Linear Regression (MLR)		
	Beta (SE)	95%CI	*p*	Beta (SE)	95%CI	*p*
Gender						
Females	reference			reference		
Males	−5.0(2.0)	−8.8, −1.1	0.012	−4.0 (1.9)	−7.9, −0.2	0.040
						
GPA						
<3	reference			reference		
≥3	2.3 (2.4)	−2.5, 7.1	0.350	1.8 (2.4)	−2.8, 6.5	0.442
						
Year						
Preclinical	reference			reference		
Clinical	−2.7 (2.2)	−7.0, 1.6	0.225	−2.5 (2.1)	−6.7, 1.7	0.248
						
Perfectionist	2.5 (0.6)	1.3, 3.7	<0.001	2.5 (0.6)	1.3, 3.7	<0.001

## Data Availability

The original contributions presented in this study are included in the article. Further inquiries can be directed to the corresponding author.
